# Supplementary material for: Novel Driver Strength Index highlights important cancer genes in TCGA PanCanAtlas patients
Source: PeerJ. 2022 Aug 11;10:e13860. doi: 10.7717/peerj.13860 (PMC9375969; doi:10.7717/peerj.13860)
Supplement: Supplemental Information 2 [file peerj-10-13860-s002.zip › COHORTS/genes plots/cohorts/2021_8_16_14_9_distribution_cohorts_females_LUSC.pdf]

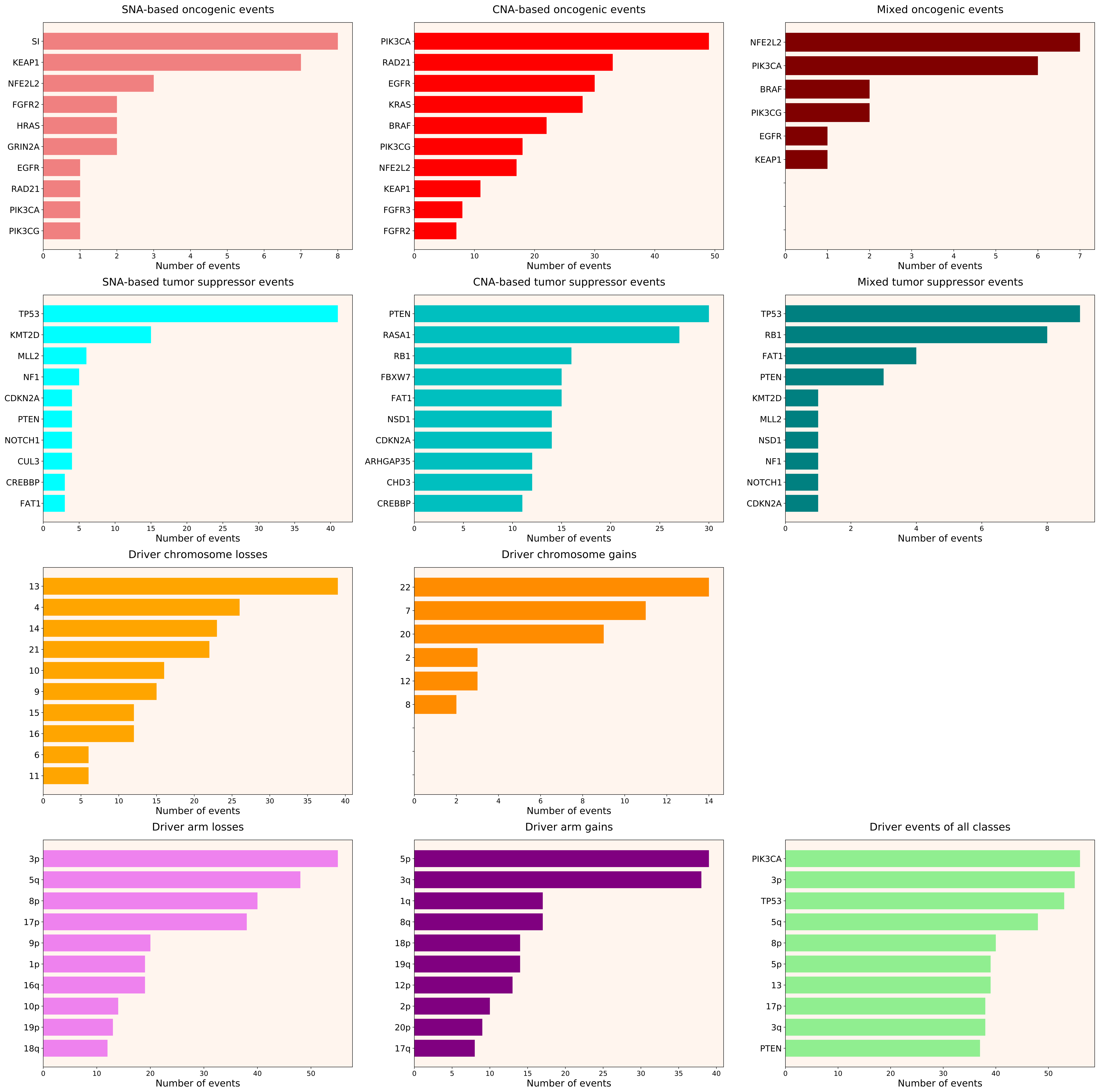

SNA-based oncogenic events
SI
KEAP1
NFE2L2
FGFR2
HRAS
GRIN2A
EGFR
RAD21
PIK3CA
PIK3CG
Number of events
CNA-based oncogenic events
PIK3CA
RAD21
EGFR
KRAS
BRAF
PIK3CG
NFE2L2
KEAP1
FGFR3
FGFR2
Number of events
Mixed oncogenic events
NFE2L2
PIK3CA
BRAF
PIK3CG
EGFR
KEAP1
Number of events
SNA-based tumor suppressor events
TP53
KMT2D
MLL2
NF1
CDKN2A
PTEN
NOTCH1
CUL3
CREBBP
FAT1
Number of events
CNA-based tumor suppressor events
PTEN
RASA1
RB1
FBXW7
FAT1
NSD1
CDKN2A
ARHGAP35
CHD3
CREBBP
Number of events
Mixed tumor suppressor events
TP53
RB1
FAT1
PTEN
KMT2D
MLL2
NSD1
NF1
NOTCH1
CDKN2A
Number of events
Driver chromosome losses
13
4
14
21
10
9
15
16
6
11
Number of events
Driver chromosome gains
22
7
20
2
12
8
Number of events
Driver arm losses
3p
5q
8p
17p
9p
1p
16q
10p
19p
18q
Number of events
Driver arm gains
5p
3q
1q
8q
18p
19q
12p
2p
20p
17q
Number of events
Driver events of all classes
PIK3CA
3p
TP53
5q
8p
5p
13
17p
3q
PTEN
Number of events
